# Supplementary material for: United Kingdom Early Detection Initiative (UK-EDI): protocol for establishing a national multicentre cohort of individuals with new-onset diabetes for early detection of pancreatic cancer
Source: BMJ Open. 2022 Oct 10;12(10):e068010. doi: 10.1136/bmjopen-2022-068010 (PMC9557307; doi:10.1136/bmjopen-2022-068010)
Supplement: Author's manuscript [file bmjopen-2022-068010.draft_revisions.pdf]

BMJ Open is committed to open peer review. As part of this commitment we make the peer review history of every article we publish publicly available.

When an article is published we post the peer reviewers' comments and the authors' responses online. We also post the versions of the paper that were used during peer review. These are the versions that the peer review comments apply to.

The versions of the paper that follow are the versions that were submitted during the peer review process. They are not the versions of record or the final published versions. They should not be cited or distributed as the published version of this manuscript.

BMJ Open is an open access journal and the full, final, typeset and author-corrected version of record of the manuscript is available on our site with no access controls, subscription charges or pay-per-view fees (http://bmjopen.bmj.com).

If you have any questions on BMJ Open's open peer review process please email info.bmjopen@bmj.com

BMJ Open

The United Kingdom Early Detection Initiative (UK-EDI): Protocol for establishing a national multi-centre cohort of individuals with new-onset diabetes for early detection of pancreatic cancer

Journal:	*BMJ Open*
Manuscript ID	bmjopen-2022-068010
Article Type:	Protocol
Date Submitted by the Author:	05-Sep-2022
Complete List of Authors:	Oldfield, Lucy; University of Liverpool, Molecular and Clinical Cancer Medicine<br>Stott, Martyn; University of Liverpool, Molecular and Clinical Cancer Medicine<br>Hanson, Robert; University of Liverpool, Liverpool Clinical Trials Centre<br>Jackson, Richard; University of Liverpool, Liverpool Clinical Trials Centre<br>Reynolds, William; University of Liverpool, Molecular and Clinical Cancer Medicine<br>Chandran-Gorner, Vatshala; University of Liverpool, Molecular and Clinical Cancer Medicine<br>Van Der Meer, Robert; University of Strathclyde, Management Science<br>Alison, Laurence; University of Liverpool<br>Tejeiro, Ricardo; University of Liverpool<br>Purewal, Tejpal; Royal Liverpool and Broadgreen Hospitals NHS Trust, Diabetes & Endocrinology<br>Ghaneh, Paula; University of Liverpool, Molecular and Clinical Cancer Medicine<br>Palmer, Daniel; University of Liverpool, Molecular and Clinical Cancer Medicine<br>Greenhalf, William; University of Liverpool<br>Halloran, Chris; University of Liverpool, Molecular and Cancer Medicine<br>Costello, Eithne; University of Liverpool, Molecular and Clinical Cancer Medicine
Keywords:	Pancreatic disease < GASTROENTEROLOGY, Diabetes & endocrinology < INTERNAL MEDICINE, Hepatobiliary tumours < ONCOLOGY

SCHOLARONE™
Manuscripts

The United Kingdom Early Detection Initiative (UK-EDI): Protocol for establishing a national multi-centre cohort of individuals with new-onset diabetes for early detection of pancreatic cancer

Authors: L. Oldfield[1∞], M. Stott[1∞], R. Hanson[2], R Jackson[2], W. Reynolds[3], V. Chandran-Görner[3], R. Van Der Meer[4], L. Alison[5], R. Tejeiro[5], T. Purewal[6], P. Ghaneh[1], D Palmer[1], W. Greenhalf[1], C. Halloran[1] and E Costello[1*].

[1]Department of Molecular and Clinical Cancer Medicine, University of Liverpool, UK

[2]Liverpool Clinical Trials Centre,

[3]GCP laboratory, University of Liverpool, UK

[4]Strathclyde Business School, University of Strathclyde, UK

[5]Department of Psychological Sciences, University of Liverpool, UK

[6]Royal Liverpool University Hospital, Prescot Street, Liverpool, UK

∞ Contributed equally to this manuscript.

*Correspondence: Eithne Costello, Department of Molecular and Clinical Cancer Medicine, University of Liverpool, Ashton Street, Liverpool L69 3GE, UK.

Email: ecostell@liverpool.ac.uk

Abstract

Introduction: Pancreatic cancer is a leading cause of cancer deaths worldwide. Screening for this disease has potential to improve survival. It is not feasible, with current screening modalities, to screen

For peer review only

the asymptomatic adult population. However, screening of individuals in high-risk groups is recommended. Our study aims to provide resources and data that will inform strategies to screen individuals with new-onset diabetes (NOD) for pancreatic cancer.

Methods and analysis: The UK-EDI study is a national, prospective, observational cohort study that aims to recruit 2,500 individuals with NOD (< 6 months post-diagnosis) aged 50 years and over, with follow-up every 6 months, over a 3-year period. For study eligibility, diagnosis of diabetes is considered to be clinical measurement of HbA1c ≥ 48 mmol/mol. Detailed clinical information and biospecimens will be collected at baseline and follow-up to support the development of molecular, epidemiological and demographic biomarkers for earlier detection of pancreatic cancer in the high-risk NOD group. Socio-economic impacts and cost-effectiveness of earlier detection of pancreatic cancer in individuals with NOD will be evaluated. The UK-EDI NOD cohort will provide a bio-resource for future early detection research to be conducted.

Ethics and dissemination: The UK-EDI study has been reviewed and approved by the London-West London and GTAC Research Ethics Committee (Ref 20/LO/0058). Study results will be disseminated through presentations at national and international symposia and publication in peer-reviewed, Open Access journals.

For peer review only

Strengths and Limitations of this study

- This study will generate the first UK cohort of individuals with new-onset diabetes, designed specifically with the intention of facilitating earlier detection of pancreatic cancer.
- The study protocol is aligned with complementary studies in the USA. The UK cohort will ensure that newly-developed early detection strategies are relevant to UK populations.
- The cohort will contain both pre-diagnostic data and biospecimens from pancreatic cancer patients and controls, allowing for the validation of existing early detection biomarkers and for future biomarker discovery.

- The study will apply health economic models to quantify the costs and benefits of detecting pancreatic cancer earlier in individuals with new-onset diabetes.
- It is anticipated that approximately 1% of the cohort of 2,500 individuals will have underlying pancreatic cancer, generating a limited number of case samples.

Introduction

Pancreatic ductal adenocarcinoma (PDAC) has the bleakest outlook in terms of survival of all common cancers. The current UK 5-year survival rate of 7.3% has improved only slightly in 40 years[1 2]. Late disease presentation is the main contributor to high mortality rates, with approximately 85% of individuals not suitable for potentially curative therapy due to locally advanced or metastatic disease. Where surgery is possible, overall survival is significantly increased[3]. Rapid intervention through earlier detection is key to improving prognosis. With a relatively low incidence rate, population-wide screening as a route to earlier detection is not justified for PDAC[4]. Screening is recommended for select high-risk groups, however, this currently represents a minority (~10%) of cases[5]. There remains a need to robustly characterise other high-risk groups for targeted screening strategies capable of capturing a larger proportion of cases [6].

Approximately 40-65% of individuals with PDAC have diabetes at the time of diagnosis[7-10], with the majority being of new-onset (< 3 years)[8 10-13]. Occurrence of diabetes in this setting is a paraneoplastic manifestation of PDAC[14], and individuals with new-onset diabetes (NOD) over the age of 50 are widely recognised as the highest risk group for PDAC[6]. The prevalence of pancreatic cancer-related diabetes (PDAC-DM) in this group is approximately 1%[15]. Consequently, screening all individuals with NOD is not feasible, as any test applied would require near perfect specificity to avoid large numbers of false positives. Methods that enrich for PDAC-DM within the group of individuals with NOD are urgently needed to aid the development of new, practical screening strategies.

PDAC-DM is a form of type 3c diabetes (T3cDM), a classification that also includes chronic pancreatitis (CP)-related diabetes, as well as other aetiologies[16-18]. T3cDM is associated with rapidly worsening glucose control and significant weight-loss[10 12 19]. Depending on study design, different estimates exist for the prevalence of T3cDM amongst those diagnosed with diabetes, ranging from 1.8 – 9.2%[16 20 21]. Molecular biomarkers along with epidemiological and clinical characteristics that enable distinction of T3cDM, or PDAC-DM, amongst NOD could facilitate screening. To-date, most studies aimed at identifying early-stage biomarkers of PDAC have used samples and associated data from patients already diagnosed with PDAC, and are thus compromised by late changes during tumorigenesis that are not seen in early stage disease. Tailor-made, pre-diagnostic cohorts are required to provide the necessary samples and associated data to support effective early detection pathways for this high-risk group.

The UK Early Detection Initiative for Pancreatic Cancer will generate a cohort of individuals with NOD, with the necessary clinical information and associated biospecimens to guide the development of a screening strategy for detection of PDAC-DM among NOD, ensuring its suitability within regional health care systems.

Methods and Analysis

Study setting

The UK-EDI Study is a national, prospective, observational cohort study, recruiting individuals with NOD aged 50 years and over to facilitate the development of screening pathways for PDAC. The study will align with a larger international effort, including studies in the United States of America[22] and the European Union. The UK-EDI study is hosted by the Liverpool Clinical Trials Centre at the University of Liverpool.

Dates of the study

From 1st April 2019 to 31st March 2024.

For peer review only

Study design

The UK-EDI Study has seven work packages (WPs) centred on the establishment of the UK-EDI cohort (WP1, Figure 1). Additional WPs include banking of blood samples to the standards of Good Clinical Practice (GCP) for laboratories (WP2), validation of existing promising biomarkers for their ability to distinguish T3cDM, including PDAC-related DM, from T2DM (WP3), interrogating epidemiological and demographic factors to further stratify risk of PDAC in the NOD population (WP4), undertaking new biomarker discovery (WP5), cost-benefit analysis (WP6), and managing and engaging stakeholders (WP7). The primary aim of the UK-EDI Study is to gather and interrogate key data to advance early detection of occult PDAC in the high-risk population of individuals with NOD.

Eligibility criteria

Eligibility criteria and relevant definitions of the UK-EDI cohort are provided in Table 1.

Table 1: Eligibility criteria and relevant definitions of the UK-EDI cohort

Criteria/definition	Details
**Inclusion criteria**	1. Aged ≥50 years at the time of study entry<br>2. New-onset diabetes (defined at HbA1c ≥ 48 mmol/mol (6.5%) diagnosed within 6 months of study entry<br>3. Willing to provide written informed consent prior to performing any protocol-related procedures<br>4. Willing and able to comply with the protocol for the duration of the study, including scheduled follow-up visits
**Exclusion criteria**	1. Diagnosis or treatment of pancreatic cancer, peri-pancreatic cancer, or pancreatic endocrine cancer<br>2. Previous surgical resection of the pancreas<br>3. Diagnosis of diabetes >6 months prior to study entry<br>4. Pregnancy

For peer review

	5. Condition preventing study investigation and follow-up
	6. Inability or incapacity to give written informed consent
**Definition of new-onset diabetes**	HbA1c ≥ 48 mmol/mol (6.5%) diagnosed within 6 months of study entry
**Identification of PDAC diagnoses**	NHS digital will be interrogated to update occurrence of PDAC

HbA1c: glycated haemoglobin, PDAC: pancreatic ductal adenocarcinoma

Methods of participant identification

The UK-EDI Study will establish a nationwide cohort representative of the UK population with recruitment occurring across primary and secondary care settings, including diabetic special centres and primary care hubs. Participants will either be identified in primary care sites and recruited in the primary care setting, or identified in primary care sites and recruited in a local secondary care recruiting hub. The use of electronic health records will facilitate six-monthly identification of suitable participants in each settings. Participants may present directly to secondary care services as emergency presentations of NOD and those individuals will be recruited in secondary care. Participants may also be identified from specialist inpatient teams such as diabetes and endocrinology, hepatobiliary and pancreatic surgery and gastroenterology. The screening framework is flexible to account for local organisation of services across the UK (Figure 2).

Study timeline

Eligible individuals will be provided with a participant information sheet explaining the UK-EDI Study, and will be given an opportunity to ask questions prior to signing an informed consent form. At the baseline visit, a full medical, drug and surgical history will be elicited including demographic, social and anthropometric data. Participants will also be asked about a range of symptoms over the preceding 6 - 12 month period, which may indicate early signs of PDAC. Participants will be asked specifically about weight changes, including in the context of a weight management programme. Quality of life assessment will be via a health-related Quality of Life Questionnaire (EQ-5D-5L) and diabetes management will be captured via a Diabetes Self-Management Questionnaire (DSMQ). The DMSQ is

For peer review only

an instrument which assesses diabetes self-care activities associated with glycaemic control. Blood samples will be taken for measurement of HbA1c and research plasma and serum samples will be taken for biobanking according to Good Clinical Practice Laboratory standards to allow for translational research. Sites may also provide the results of other haematological and biochemical blood results including full blood count, liver function tests, urea and electrolytes and lipid profiles, if these are being taken for routine diabetes care. Supplemental blood test results are not required for all participants.

Follow up visits will be at 6, 12, 18 and 24 months after the baseline visit. Quality of Life, DSMQ, and case report form data will be collected at baseline and each follow-up visit. At 36 months there will be a search of NHS Digital to determine the number of events of PDAC diagnosis in the enrolled cohort.

Key clinical and translational characteristics, including timeframes, of the UK-EDI cohort are listed in Table 2.

Table 2: Eligibility criteria and relevant definitions of the UK-EDI cohort

Data Item	Details
EDTA stored blood	Translational blood samples taken at baseline, 6, 12, 18 and 24 months stored to GCP standards
Serum stored blood	Translational blood samples taken at baseline, 6, 12, 18 and 24 months stored to GCP standards
HbA1c measurements	HbA1c measurements from diagnosis, baseline, 6, 12, 18 and 24 months
Demographics	Details including ethnicity, smoking, and alcohol status
Anthropometric data	Height, weight, waist and hip measurements taken at baseline, 6, 12, 18 and 24 months
Biochemistry and symptomology of diabetes onset	Details regarding symptoms at the time of diabetes diagnosis and prior biochemical (HbA1c) data pre-diagnosis (left-window)

For peer review only

**Symptomology relevant to pancreatic cancer**	Detailed information regarding symptoms typical of onset of pancreatic cancer, and changes from baseline at 6, 12, 18 and 24 months
**DSMQ**	The diabetes self-management questionnaire. A validated instrument assessing self-care behaviours associated with diabetes control. Measured at baseline, 6, 12, 18 and 24 months
**EQ-5D-5L**	Standardised measure of health-related quality of life measured at baseline, 6, 12, 18 and 24 months
**Medical and surgical history**	Changes to medical and surgical diagnoses at baseline, 6, 12, 18 and 24 months
**Medication history**	Changes to prescribed medication, including for diabetes, at baseline, 6, 12, 18 and 24 months
**Pancreatic malignancy data**	Data from subsequent pancreatic cancer diagnoses, including surgical and oncological therapy.

GCP: Good Clinical Practice, HbA1c: glycated haemoglobin, DSMQ; Diabetes Self-Management Questionnaire, EQ-5D-5L: Quality of Life Questionnaire

Objectives

Primary

The primary objective is to recruit individuals to a bespoke standardised cohort of individuals aged 50 years or older with NOD (HbA1C ≥ 48 mmol/mol, (6.5%)) and no prior history of DM, ensuring the standardised collection and biobanking of samples whilst acquiring the molecular, epidemiological and demographic factors in order to advance the early detection of PDAC.

Secondary

The secondary objectives are to validate CA19-9 and other novel markers already identified with potential to distinguish T3cDM (including PDAC-related) from T2DM, and to establish the economic impact of diagnosing PDAC early in individuals with NOD.

Exploratory

For peer review only

The opportunistic and exploratory objectives are to study molecular, epidemiological and demographic factors to further stratify risk of PDAC and to use the UK-EDI cohort for biomarker discovery.

Statistical methodology

Sample Size

We aim to recruit 2,500 patients with NOD aged 50 years and older, with a follow up of 36 months. The target size of 2,500 is pragmatic, based on costs and what is practically possible. It is anticipated that 0.8-1% of the group will receive a diagnosis of PDAC in the three-year follow-up time period[15].

Thus, the UK-EDI cohort is expected to yield approximately 21 to 25 PDAC diagnoses (or 17 to 20 cases with 20% attrition). This study will provide data on the incidence of PDAC in individuals with NOD in the UK, and will serve as the benchmark/reference point for future work.

Formal power calculations to determine associations between clinical/biological subgroups and the diagnosis of PDAC are difficult as it is not yet known what fraction of any subgroups will split the available patient population.

The UK-EDI cohort will contribute to larger international efforts aimed at determining the feasibility of detecting resectable PDAC in individuals over 50 years of age who are newly diagnosed with diabetes mellitus[23].

Data analysis plan

The primary endpoint of interest is clinically diagnosed PDAC within 3 years of a new diagnosis of DM. The measurement of clinical characteristics, including glucose control (HbA1c) along with biological and epidemiological measures at five time points over the course of the cohort study will help inform the incidence of PDAC in individuals with NOD in the UK.

Formal power calculations to determine associations between clinical or biological subgroups and the diagnosis of PDAC are difficult as it is not yet known what fraction of any subgroups will split the available patient population. Analysis of PDAC diagnosis will be performed using longitudinal methods,

For peer review only

assessing the effect of biological and epidemiological markers whilst adjusting for relevant clinical characteristics.

Validation of biomarkers will include receiver operator characteristic (ROC) curve analysis to determine biomarker performance characteristics. Further exploratory analysis will be carried out using multivariate techniques such as principal component and hierarchical cluster analyses in order to reduce the dimensionality of the data and identify naturally forming groups within the data respectively.

Secondary analyses will focus on the time to detection of PDAC using a time-to-event approach. The probability of detecting PDAC will be calculated across subgroups using the method of Kaplan Meier. Inclusion of biological and clinical characteristics will be incorporated using joint survival/longitudinal modelling techniques. Detailed information including clinical, epidemiological and biomarker data will be used to build a PDAC risk score, or validate emerging risk scores.

To assess the cost-effectiveness of early diagnosis of PDAC in individuals with NOD, the clinical pathway will be mapped and a literature review conducted to ensure that the model is populated with relevant current data. The study questionnaires will be used to update and calibrate models with data pertaining to PDAC cases versus controls. At the cohort level, a Markov model will be constructed to incorporate assumptions about what would happen to patients if they were identified earlier. In addition, a discrete event simulation model will be developed to capture stochastic variations at the level of individual patients. The impact of early diagnosis will be analysed using the updated Markov and simulation models. With respect to patients detected earlier in a future screening protocol implemented in NOD, a key uncertainty is the stage of disease that would be diagnosed. A probabilistic sensitivity analysis, informed by the literature, will be conducted on a range of scenarios, including epidemiological, clinical and biomarker data.

Patient and public involvement statement

The Liverpool Pancreatic Patient and Public Involvement Group have contributed to the study conception and design and they have continued involvement in the management of this study. There

For peer review only

are lay representatives on the Trial Steering Committee to ensure the study remains both acceptable and relevant to patients.

Ethics and Dissemination

Ethical approval

UK-EDI is approved by the UK Health Research Authority with favourable opinion granted by the London-West London and GTAC Research Ethics Committee on 14th February 2020. The details of this manuscript represent Version 5 of the protocol approved on the 9th September 2021.

The study will be conducted in accordance with the Human Rights Act 1998, the Data Protection Act 2018, Freedom of Information Act 2000, the principles of Good Clinical Practice, the Declaration of Helsinki on biomedical research involving human volunteers (Hong Kong revision, 1989 and the 48th General Assembly, Somerset West, Republic of South Africa, October 1996, updated in October 2013) and the UK Policy Framework for Health and Social Care Research. Where individuals agree to take part in the study, they will be informed of how data are recorded, collected, stored and processed, and that data may be transferred to other countries, in accordance with UK General Data Protection Regulations (UK GDPR).

Data Management considerations

Data Management will be through the Liverpool Clinical Trials Centre with delegated responsibilities for the University of Liverpool. The study has a dedicated Trial Manager, Data Manager and Trial Statistician and will be overseen by a Trial Management Committee and Trial Steering Committee. Data Management will be through the REDCap database and a dedicated Laboratory Information Management System (LIMS) within the Good Clinical Practice Laboratories at the University of Liverpool, linked by unique codes for each kit used at each time point from each patient, the code for which will be stored on REDCap and LIMS.

Dissemination plan

For peer review only

Study results will be disseminated through presentations at national and international symposia and publication in peer-reviewed, Open Access journals. Where appropriate data will be made available via open access repositories. We will work with charities, patient and public involvement groups, and other relevant stakeholders to widely disseminate results and ensure that our findings are in an accessible format.

Acknowledgements

The authors are grateful for the support of all participants contributing to this study.

Authors' contributions

EC, LO, WG, CH, RVDM, LA, RT and TP conceived and designed the study. LO, MS and EC drafted this manuscript. RH, MS, EC, WG, RJ and CH drafted the study protocol. RJ is responsible for statistical design and analysis. EC is chief investigator. All authors contributed to the final manuscript and agreed to all of the content of the submitted manuscript.

Funding statement

This work is supported by Cancer Research UK, grant number C7690/A26881.

Competing interests statement

EC, LO, WG, CH and PG are named as inventors on GB patent GBGB1806002.0; PCT/GB2019/050998, submitted by the University of Liverpool, that covers the measurement of adiponectin and IL-1Ra as a biomarker for early detection of pancreatic cancer. No other competing interests declared.

References

1. Sung H, Ferlay J, Siegel RL, et al. Global Cancer Statistics 2020: GLOBOCAN Estimates of Incidence and Mortality Worldwide for 36 Cancers in 185 Countries. *CA: a cancer journal for clinicians* 2021;71(3):209-49. doi: 10.3322/caac.21660 [published Online First: 2021/02/05]
2. Office for National Statistics. Cancer survival in England 2013 - 2017. ONS website; 12 August 2019: Accessed June 2022
3. Neoptolemos JP, Palmer DH, Ghaneh P, et al. Comparison of adjuvant gemcitabine and capecitabine with gemcitabine monotherapy in patients with resected pancreatic cancer (ESPAC-4): a multicentre, open-label, randomised, phase 3 trial. Lancet 2017;389(10073):1011-24. doi: 10.1016/s0140-6736(16)32409-6 [published Online First: 2017/01/29]

For peer review only

4. Owens DK, Davidson KW, Krist AH, et al. Screening for Pancreatic Cancer: US Preventive Services Task Force Reaffirmation Recommendation Statement. *Jama* 2019;322(5):438-44. doi: 10.1001/jama.2019.10232 [published Online First: 2019/08/07]
5. Greenhalf W, Grocock C, Harcus M, et al. Screening of high-risk families for pancreatic cancer. *Pancreatology* 2009;9(3):215-22. doi: 10.1159/000210262 [published Online First: 2009/04/08]
6. Pereira SP, Oldfield L, Ney A, et al. Early detection of pancreatic cancer. *The Lancet Gastroenterology & Hepatology* 2020 doi: https://doi.org/10.1016/S2468-1253(19)30416-9
7. Pannala R, Leirness JB, Bamlet WR, et al. Prevalence and clinical profile of pancreatic cancer-associated diabetes mellitus. *Gastroenterology* 2008;134(4):981-87. doi: 10.1053/j.gastro.2008.01.039
8. Aggarwal G, Kamada P, Chari ST. Prevalence of diabetes mellitus in pancreatic cancer compared to common cancers. *Pancreas* 2013;42(2):198-201. doi: 10.1097/MPA.0b013e3182592c96 [published Online First: 2012/09/25]
9. Ose DJ, Viskochil R, Holowatyj AN, et al. Understanding the Prevalence of Prediabetes and Diabetes in Patients With Cancer in Clinical Practice: A Real-World Cohort Study. *Journal of the National Comprehensive Cancer Network : JNCCN* 2021;19(6):709-18. doi: 10.6004/jnccn.2020.7653 [published Online First: 2021/06/16]
10. Brewer MJ, Doucette JT, Bar-Mashiah A, et al. Glycemic Changes and Weight Loss Precede Pancreatic Ductal Adenocarcinoma by up to 3 Years in a Diverse Population. *Clin Gastroenterol Hepatol* 2022;20(5):1105-11.e2. doi: 10.1016/j.cgh.2021.07.046 [published Online First: 2021/08/07]
11. Mueller AM, Meier CR, Jick SS, et al. Characterization of the deterioration of diabetes control in patients with a subsequent diagnosis of pancreatic cancer: A descriptive study. *Pancreatology* 2022;22(3):387-95. doi: 10.1016/j.pan.2022.03.012 [published Online First: 2022/03/23]
12. Tan PS, Garriga C, Clift A, et al. Temporality of body mass index, blood tests, comorbidities and medication use as early markers for pancreatic ductal adenocarcinoma (PDAC): a nested case-control study. *Gut* 2022 doi: 10.1136/gutjnl-2021-326522 [published Online First: 2022/06/28]
13. Chari ST, Leibson CL, Rabe KG, et al. Pancreatic cancer-associated diabetes mellitus: Prevalence and temporal association with diagnosis of cancer. *Gastroenterology* 2008;134(1):95-101. doi: 10.1053/j.gastro.2007.10.040
14. Andersen DK, Korc M, Petersen GM, et al. Diabetes, Pancreatogenic Diabetes, and Pancreatic Cancer. *Diabetes* 2017;66(5):1103-10. doi: 10.2337/db16-1477 [published Online First: 2017/05/17]
15. Chari ST, Leibson CL, Rabe KG, et al. Probability of pancreatic cancer following diabetes: A population-based study. *Gastroenterology* 2005;129(2):504-11. doi: 10.1053/j.gastro.2005.05.007
16. Woodmansey C, McGovern AP, McCullough KA, et al. Incidence, Demographics, and Clinical Characteristics of Diabetes of the Exocrine Pancreas (Type 3c): A Retrospective Cohort Study. *Diabetes Care* 2017;40(11):1486-93. doi: 10.2337/dc17-0542 [published Online First: 2017/09/02]
17. Cui Y, Andersen DK. Pancreatogenic diabetes: special considerations for management. *Pancreatology* 2011;11(3):279-94. doi: 10.1159/000329188 [published Online First: 2011/07/16]
18. American Diabetes Association. 2. Classification and Diagnosis of Diabetes: Standards of Medical Care in Diabetes—2020. *Diabetes Care* 2019;43(Supplement_1):S14-S31. doi: 10.2337/dc20-S002
19. Hart PA, Kamada P, Rabe KG, et al. Weight Loss Precedes Cancer-Specific Symptoms in Pancreatic Cancer-Associated Diabetes Mellitus. *Pancreas* 2011;40(5):768-72. doi: 10.1097/MPA.0b013e318220816a

For peer review only

20. Vujasinovic M, Zaletel J, Tepes B, et al. Low prevalence of exocrine pancreatic insufficiency in patients with diabetes mellitus. *Pancreatology* 2013;13(4):343-6. doi: 10.1016/j.pan.2013.05.010 [published Online First: 2013/07/31]
21. Ewald N, Kaufmann C, Raspe A, et al. Prevalence of diabetes mellitus secondary to pancreatic diseases (type 3c). *Diabetes-Metabolism Research and Reviews* 2012;28(4):338-42. doi: 10.1002/dmrr.2260
22. Maitra A, Sharma A, Brand RE, et al. A Prospective Study to Establish a New-Onset Diabetes Cohort: From the Consortium for the Study of Chronic Pancreatitis, Diabetes, and Pancreatic Cancer. *Pancreas* 2018;47(10):1244-48. doi: 10.1097/MPA.0000000000001169 [published Online First: 2018/10/17]
23. Chari ST, Maitra A, Matrisian LM, et al. Early Detection Initiative: A randomized controlled trial of algorithm-based screening in patients with new onset hyperglycemia and diabetes for early detection of pancreatic ductal adenocarcinoma. *Contemporary clinical trials* 2022;113:106659. doi: 10.1016/j.cct.2021.106659 [published Online First: 2021/12/27]

For peer review only

Figure legends

Figure 1: UK Early Detection Initiative for Pancreatic Cancer (UK-EDI) Programme outline. *NOD: new-onset diabetes mellitus; PDAC: pancreatic ductal adenocarcinoma; T2DM: type 2 diabetes mellitus; T3cDM: type 3c diabetes mellitus; WP: work package.*

Figure 2: Schematic of UK-EDI study recruitment and participant follow-up. *HbA1c: glycated haemoglobin; EMIS: Egton Medical Information Systems (electronic healthcare record system)*

Word count (excluding abstract, figures and tables): 3107

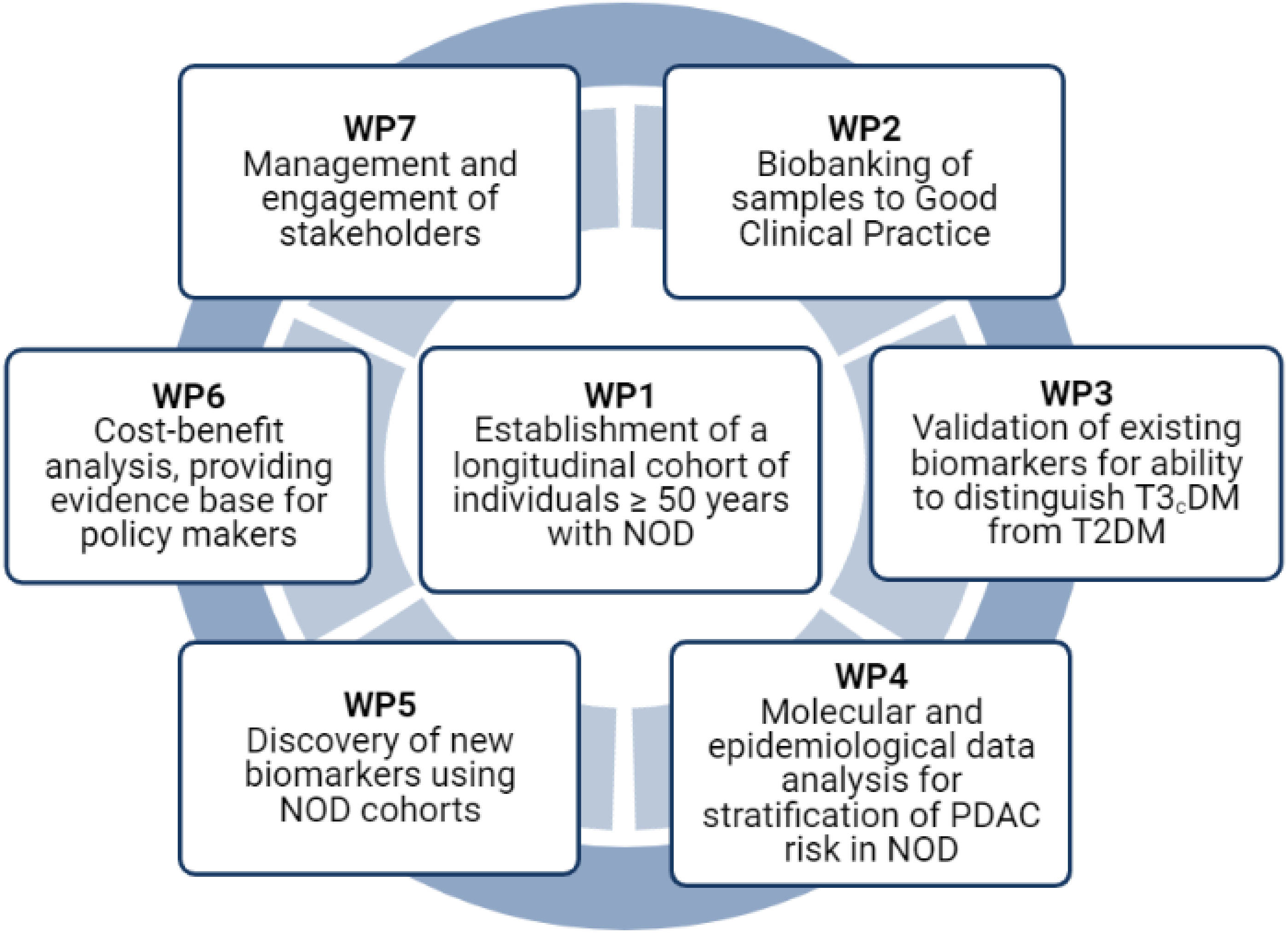
WP7
Management and engagement of stakeholders
WP2
Biobanking of samples to Good Clinical Practice
WP6
Cost-benefit analysis, providing evidence base for policy makers
WP1
Establishment of a longitudinal cohort of individuals ≥ 50 years with NOD
WP3
Validation of existing biomarkers for ability to distinguish T3cDM from T2DM
WP5
Discovery of new biomarkers using NOD cohorts
WP4
Molecular and epidemiological data analysis for stratification of PDAC risk in NOD

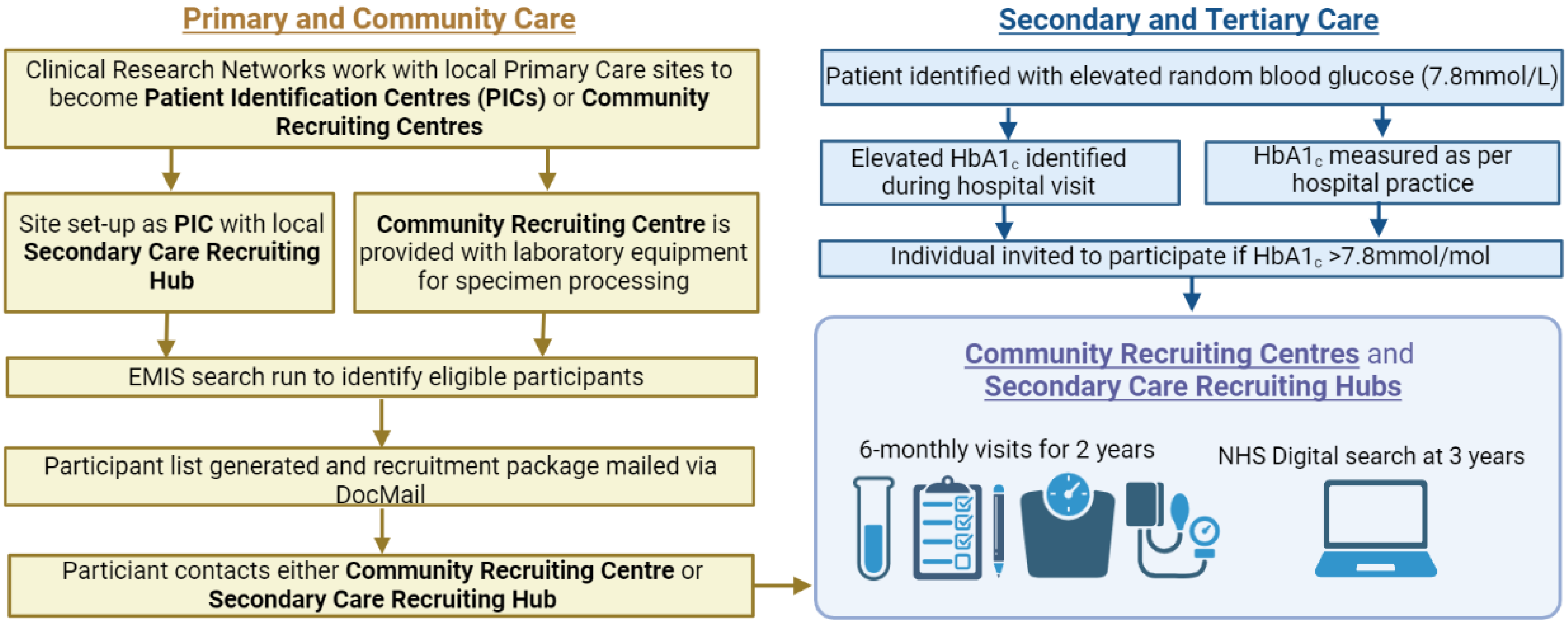
Primary and Community Care
Clinical Research Networks work with local Primary Care sites to become Patient Identification Centres (PICs) or Community Recruiting Centres
Site set-up as PIC with local Secondary Care Recruiting Hub
Community Recruiting Centre is provided with laboratory equipment for specimen processing
EMIS search run to identify eligible participants
Participant list generated and recruitment package mailed via DocMail
Particiant contacts either Community Recruiting Centre or Secondary Care Recruiting Hub
Secondary and Tertiary Care
Patient identified with elevated random blood glucose (7.8mmol/L)
Elevated HbA1c identified during hospital visit
HbA1c measured as per hospital practice
Individual invited to participate if HbA1c >7.8mmol/mol
Community Recruiting Centres and Secondary Care Recruiting Hubs
6-monthly visits for 2 years
NHS Digital search at 3 years

BMJ Open

The United Kingdom Early Detection Initiative (UK-EDI): Protocol for establishing a national multi-centre cohort of individuals with new-onset diabetes for early detection of pancreatic cancer

Journal:	*BMJ Open*
Manuscript ID	bmjopen-2022-068010.R1
Article Type:	Protocol
Date Submitted by the Author:	20-Sep-2022
Complete List of Authors:	Oldfield, Lucy; University of Liverpool, Molecular and Clinical Cancer Medicine<br>Stott, Martyn; University of Liverpool, Molecular and Clinical Cancer Medicine<br>Hanson, Robert; University of Liverpool, Liverpool Clinical Trials Centre<br>Jackson, Richard; University of Liverpool, Liverpool Clinical Trials Centre<br>Reynolds, William; University of Liverpool, Molecular and Clinical Cancer Medicine<br>Chandran-Gorner, Vatshala; University of Liverpool, Molecular and Clinical Cancer Medicine<br>Van Der Meer, Robert; University of Strathclyde, Management Science<br>Alison, Laurence; University of Liverpool<br>Tejeiro, Ricardo; University of Liverpool<br>Purewal, Tejpal; Royal Liverpool and Broadgreen Hospitals NHS Trust, Diabetes & Endocrinology<br>Ghaneh, Paula; University of Liverpool, Molecular and Clinical Cancer Medicine<br>Palmer, Daniel; University of Liverpool, Molecular and Clinical Cancer Medicine<br>Greenhalf, William; University of Liverpool<br>Halloran, Chris; University of Liverpool, Molecular and Cancer Medicine<br>Costello, Eithne; University of Liverpool, Molecular and Clinical Cancer Medicine
**Primary Subject Heading**:	Gastroenterology and hepatology
Secondary Subject Heading:	Oncology, General practice / Family practice
Keywords:	Pancreatic disease < GASTROENTEROLOGY, Diabetes & endocrinology < INTERNAL MEDICINE, Hepatobiliary tumours < ONCOLOGY

SCHOLARONE™ Manuscripts

The United Kingdom Early Detection Initiative (UK-EDI): Protocol for establishing a national multi-centre cohort of individuals with new-onset diabetes for early detection of pancreatic cancer

Authors: L. Oldfield[1∞], M. Stott[1∞], R. Hanson[2], R Jackson[2], W. Reynolds[3], V. Chandran-Görner[3], R. Van Der Meer[4], L. Alison[5], R. Tejeiro[5], T. Purewal[6], P. Ghaneh[1], D Palmer[1], W. Greenhalf[1], C. Halloran[1] and E Costello[1*].

[1]Department of Molecular and Clinical Cancer Medicine, University of Liverpool, UK

[2]Liverpool Clinical Trials Centre,

[3]GCP laboratory, University of Liverpool, UK

[4]Strathclyde Business School, University of Strathclyde, UK

[5]Department of Psychological Sciences, University of Liverpool, UK

[6]Royal Liverpool University Hospital, Prescot Street, Liverpool, UK

∞ Contributed equally to this manuscript.

*Correspondence: Eithne Costello, Department of Molecular and Clinical Cancer Medicine, University of Liverpool, Ashton Street, Liverpool L69 3GE, UK.

Email: ecostell@liverpool.ac.uk

For peer review only

Abstract

Introduction: Pancreatic cancer is a leading cause of cancer deaths worldwide. Screening for this disease has potential to improve survival. It is not feasible, with current screening modalities, to screen

the asymptomatic adult population. However, screening of individuals in high-risk groups is recommended. Our study aims to provide resources and data that will inform strategies to screen individuals with new-onset diabetes (NOD) for pancreatic cancer.

Methods and analysis: The UK-EDI study is a national, prospective, observational cohort study that aims to recruit 2,500 individuals with NOD (< 6 months post-diagnosis) aged 50 years and over, with follow-up every 6 months, over a 3-year period. For study eligibility, diagnosis of diabetes is considered to be clinical measurement of HbA1c ≥ 48 mmol/mol. Detailed clinical information and biospecimens will be collected at baseline and follow-up to support the development of molecular, epidemiological and demographic biomarkers for earlier detection of pancreatic cancer in the high-risk NOD group. Socio-economic impacts and cost-effectiveness of earlier detection of pancreatic cancer in individuals with NOD will be evaluated. The UK-EDI NOD cohort will provide a bio-resource for future early detection research to be conducted.

Ethics and dissemination: The UK-EDI study has been reviewed and approved by the London-West London and GTAC Research Ethics Committee (Ref 20/LO/0058). Study results will be disseminated through presentations at national and international symposia and publication in peer-reviewed, Open Access journals.

Strengths and Limitations of this study

- UK-EDI will generate the first UK cohort of individuals with new-onset diabetes, designed specifically with the intention of facilitating earlier detection of pancreatic cancer.
- The study is designed to obtain pre-diagnostic data and biospecimens from pancreatic cancer patients and controls.
- Pre-diagnostic samples and data will be generated for the validation of existing early detection biomarkers and for future biomarker discovery.

For peer review only

- The study will apply health economic models to quantify the costs and benefits of detecting pancreatic cancer earlier in individuals with new-onset diabetes.
- It is anticipated that approximately 1% of the cohort of 2,500 individuals will have underlying pancreatic cancer, generating a limited number of case samples.

Introduction

Pancreatic ductal adenocarcinoma (PDAC) has the bleakest outlook in terms of survival of all common cancers. The current UK 5-year survival rate of 7.3% has improved only slightly in 40 years[1 2]. Late disease presentation is the main contributor to high mortality rates, with approximately 85% of individuals not suitable for potentially curative therapy due to locally advanced or metastatic disease. Where surgery is possible, overall survival is significantly increased[3]. Rapid intervention through earlier detection is key to improving prognosis. With a relatively low incidence rate, population-wide screening as a route to earlier detection is not justified for PDAC[4]. Screening is recommended for select high-risk groups, however, this currently represents a minority (~10%) of cases[5]. There remains a need to robustly characterise other high-risk groups for targeted screening strategies capable of capturing a larger proportion of cases [6].

Approximately 40-65% of individuals with PDAC have diabetes at the time of diagnosis[7-10], with the majority being of new-onset (< 3 years)[8 10-13]. Occurrence of diabetes in this setting is a paraneoplastic manifestation of PDAC[14], and individuals with new-onset diabetes (NOD) over the age of 50 are widely recognised as the highest risk group for PDAC[6]. The prevalence of pancreatic cancer-related diabetes (PDAC-DM) in this group is approximately 1%[15]. Consequently, screening all individuals with NOD is not feasible, as any test applied would require near perfect specificity to avoid large numbers of false positives. Methods that enrich for PDAC-DM within the group of individuals with NOD are urgently needed to aid the development of new, practical screening strategies.

PDAC-DM is a form of type 3c diabetes (T3cDM), a classification that also includes chronic pancreatitis (CP)-related diabetes, as well as other aetiologies[16-18]. T3cDM is associated with rapidly worsening glucose control and significant weight-loss[10 12 19]. Depending on study design, different estimates exist for the prevalence of T3cDM amongst those diagnosed with diabetes, ranging from 1.8 – 9.2%[16 20 21]. Molecular biomarkers along with epidemiological and clinical characteristics that enable distinction of T3cDM, or PDAC-DM, amongst NOD could facilitate screening. To-date, most studies aimed at identifying early-stage biomarkers of PDAC have used samples and associated data from patients already diagnosed with PDAC, and are thus compromised by late changes during tumorigenesis that are not seen in early stage disease. Tailor-made, pre-diagnostic cohorts are required to provide the necessary samples and associated data to support effective early detection pathways for this high-risk group.

The UK Early Detection Initiative for Pancreatic Cancer will generate a cohort of individuals with NOD, with the necessary clinical information and associated biospecimens to guide the development of a screening strategy for detection of PDAC-DM among NOD, ensuring its suitability within regional health care systems.

Methods and Analysis

Study setting

The UK-EDI Study is a national, prospective, observational cohort study, recruiting individuals with NOD aged 50 years and over to facilitate the development of screening pathways for PDAC. The study will align with a larger international effort, including studies in the United States of America[22] and the European Union. The UK-EDI study is hosted by the Liverpool Clinical Trials Centre at the University of Liverpool.

Dates of the study

From 18th January 2021 to 31st March 2024.

For peer review only

Study design

The UK-EDI Study has seven work packages (WPs) centred on the establishment of the UK-EDI cohort (WP1, Figure 1). Additional WPs include banking of blood samples to the standards of Good Clinical Practice (GCP) for laboratories (WP2), validation of existing promising biomarkers for their ability to distinguish T3cDM, including PDAC-related DM, from T2DM (WP3), interrogating epidemiological and demographic factors to further stratify risk of PDAC in the NOD population (WP4), undertaking new biomarker discovery (WP5), cost-benefit analysis (WP6), and managing and engaging stakeholders (WP7). The primary aim of the UK-EDI Study is to gather and interrogate key data to advance early detection of occult PDAC in the high-risk population of individuals with NOD.

For pragmatic reasons, the UK-EDI study does not contain an imaging component. A similar trial underway in the United States, designed to improve detection of operable PDAC in individuals with NOD, includes an imaging arm[23]. In that study, the Enriching New-onset Diabetes for Pancreatic Cancer (ENDPAC) algorithm risk stratifies individuals with NOD based on age, and changes in both weight and diabetes parameters[23]. Individuals with high ENDPAC scores are stratified to the intervention arm.

Eligibility criteria

Eligibility criteria and relevant definitions of the UK-EDI cohort are provided in Table 1.

Table 1: Eligibility criteria and relevant definitions of the UK-EDI cohort

Criteria/definition	Details
**Inclusion criteria**	1. Aged ≥50 years at the time of study entry<br>2. New-onset diabetes (defined at HbA1c ≥ 48 mmol/mol (6.5%) diagnosed within 6 months of study entry<br>3. Willing to provide written informed consent prior to performing any protocol-related procedures

For peer review only

	4. Willing and able to comply with the protocol for the duration of the study, including scheduled follow-up visits
**Exclusion criteria**	1. Diagnosis or treatment of pancreatic cancer, peri-pancreatic cancer, or pancreatic endocrine cancer<br>2. Previous surgical resection of the pancreas<br>3. Diagnosis of diabetes >6 months prior to study entry<br>4. Pregnancy<br>5. Condition preventing study investigation and follow-up<br>6. Inability or incapacity to give written informed consent
**Definition of new-onset diabetes**	HbA1c ≥ 48 mmol/mol (6.5%) diagnosed within 6 months of study entry
**Identification of PDAC diagnoses**	NHS digital will be interrogated to update occurrence of PDAC

HbA1c: glycated haemoglobin, PDAC: pancreatic ductal adenocarcinoma

Methods of participant identification

The UK-EDI Study will establish a nationwide cohort representative of the UK population with recruitment occurring across primary and secondary care settings, including diabetic special centres and primary care hubs. In an internal pilot study, we established pathways for identification of individuals with NOD from primary care, with recruitment in secondary care[24] In the UK-EDI study, participants will either be identified in primary care sites and recruited in the primary care setting, or identified in primary care sites and recruited in a local secondary care recruiting hub.. The use of electronic health records will facilitate six-monthly identification of suitable participants in each settings. Participants may present directly to secondary care services as emergency presentations of NOD and those individuals will be recruited in secondary care. Participants may also be identified from specialist inpatient teams such as diabetes and endocrinology, hepatobiliary and pancreatic surgery and gastroenterology. The screening framework is flexible to account for local organisation of services across the UK (Figure 2).

Study timeline

For peer review only

Eligible individuals will be provided with a participant information sheet explaining the UK-EDI Study, and will be given an opportunity to ask questions prior to signing an informed consent form. At the baseline visit, a full medical, drug and surgical history will be elicited including demographic, social and anthropometric data. Participants will also be asked about a range of symptoms over the preceding 6 - 12 month period, which may indicate early signs of PDAC. Participants will be asked specifically about weight changes, including in the context of a weight management programme. Quality of life assessment will be via a health-related Quality of Life Questionnaire (EuroQol Research Foundation: EQ-5D-5L)[25] and diabetes management will be captured via a Diabetes Self-Management Questionnaire (DSMQ)[26]. The DMSQ is an instrument which assesses diabetes self-care activities associated with glycaemic control. Blood samples will be taken for measurement of HbA1c and research plasma and serum samples will be taken for biobanking according to Good Clinical Practice Laboratory standards to allow for translational research. Sites may also provide the results of other haematological and biochemical blood results including full blood count, liver function tests, urea and electrolytes and lipid profiles, if these are being taken for routine diabetes care. Supplemental blood test results are not required for all participants.

Follow up visits will be at 6, 12, 18 and 24 months after the baseline visit. Quality of Life, DSMQ, and case report form data will be collected at baseline and each follow-up visit. At 36 months there will be a search of NHS Digital to determine the number of events of PDAC diagnosis in the enrolled cohort.

Key clinical and translational characteristics, including timeframes, of the UK-EDI cohort are listed in Table 2.

Table 2: Eligibility criteria and relevant definitions of the UK-EDI cohort

Data Item	Details
**EDTA stored blood**	Translational blood samples taken at baseline, 6, 12, 18 and 24 months stored to GCP standards

For peer review only

**Serum stored blood**	Translational blood samples taken at baseline, 6, 12, 18 and 24 months stored to GCP standards
**HbA1c measurements**	HbA1c measurements from diagnosis, baseline, 6, 12, 18 and 24 months
**Demographics**	Details including ethnicity, smoking, and alcohol status
**Anthropometric data**	Height, weight, waist and hip measurements taken at baseline, 6, 12, 18 and 24 months
**Biochemistry and symptomology of diabetes onset**	Details regarding symptoms at the time of diabetes diagnosis and prior biochemical (HbA1c) data pre-diagnosis (left-window)
**Symptomology relevant to pancreatic cancer**	Detailed information regarding symptoms typical of onset of pancreatic cancer, and changes from baseline at 6, 12, 18 and 24 months
**DSMQ**	The diabetes self-management questionnaire. A validated instrument assessing self-care behaviours associated with diabetes control. Measured at baseline, 6, 12, 18 and 24 months
**EQ-5D-5L**	Standardised measure of health-related quality of life measured at baseline, 6, 12, 18 and 24 months
**Medical and surgical history**	Changes to medical and surgical diagnoses at baseline, 6, 12, 18 and 24 months
**Medication history**	Changes to prescribed medication, including for diabetes, at baseline, 6, 12, 18 and 24 months
**Pancreatic malignancy data**	Data from subsequent pancreatic cancer diagnoses, including surgical and oncological therapy.

GCP: Good Clinical Practice, HbA1c: glycated haemoglobin, DSMQ; Diabetes Self-Management Questionnaire, EQ-5D-5L: EuroQol Research Foundation Quality of Life Questionnaire

Objectives

Primary

The primary objective is to recruit individuals to a bespoke standardised cohort of individuals aged 50 years or older with NOD (HbA1C ≥ 48 mmol/mol, (6.5%)) and no prior history of DM, ensuring the

For peer review only

standardised collection and biobanking of samples whilst acquiring the molecular, epidemiological and demographic factors in order to advance the early detection of PDAC.

Secondary

The secondary objectives are to validate CA19-9 and other novel markers already identified with potential to distinguish T3cDM (including PDAC-related) from T2DM, and to establish the economic impact of diagnosing PDAC early in individuals with NOD.

Exploratory

The opportunistic and exploratory objectives are to study molecular, epidemiological and demographic factors to further stratify risk of PDAC and to use the UK-EDI cohort for biomarker discovery.

Statistical methodology

Sample Size

We aim to recruit 2,500 patients with NOD aged 50 years and older, with a follow up of 36 months. The target size of 2,500 is pragmatic, based on costs and what is practically possible. It is anticipated that 0.8-1% of the group will receive a diagnosis of PDAC in the three-year follow-up time period[15].

Thus, the UK-EDI cohort is expected to yield approximately 21 to 25 PDAC diagnoses (or 17 to 20 cases with 20% attrition). This study will provide data on the incidence of PDAC in individuals with NOD in the UK, and will serve as the benchmark/reference point for future work.

Formal power calculations to determine associations between clinical/biological subgroups and the diagnosis of PDAC are difficult as it is not yet known what fraction of any subgroups will split the available patient population.

The UK-EDI cohort will contribute to larger international efforts aimed at determining the feasibility of detecting resectable PDAC in individuals over 50 years of age who are newly diagnosed with diabetes mellitus[23].

Data analysis plan

For peer review only

The primary endpoint of interest is clinically diagnosed PDAC within 3 years of a new diagnosis of DM. The measurement of clinical characteristics, including glucose control (HbA1c) along with biological and epidemiological measures at five time points over the course of the cohort study will help inform the incidence of PDAC in individuals with NOD in the UK.

Formal power calculations to determine associations between clinical or biological subgroups and the diagnosis of PDAC are difficult as it is not yet known what fraction of any subgroups will split the available patient population. Analysis of PDAC diagnosis will be performed using longitudinal methods, assessing the effect of biological and epidemiological markers whilst adjusting for relevant clinical characteristics.

Validation of biomarkers will include receiver operator characteristic (ROC) curve analysis to determine biomarker performance characteristics. Further exploratory analysis will be carried out using multivariate techniques such as principal component and hierarchical cluster analyses in order to reduce the dimensionality of the data and identify naturally forming groups within the data respectively.

Secondary analyses will focus on the time to detection of PDAC using a time-to-event approach. The probability of detecting PDAC will be calculated across subgroups using the method of Kaplan Meier. Inclusion of biological and clinical characteristics will be incorporated using joint survival/longitudinal modelling techniques. Detailed information including clinical, epidemiological and biomarker data will be used to build a PDAC risk score, or validate emerging risk scores.

To assess the cost-effectiveness of early diagnosis of PDAC in individuals with NOD, the clinical pathway will be mapped and a literature review conducted to ensure that the model is populated with relevant current data. The study questionnaires will be used to update and calibrate models with data pertaining to PDAC cases versus controls. At the cohort level, a Markov model will be constructed to incorporate assumptions about what would happen to patients if they were identified earlier. In addition, a discrete event simulation model will be developed to capture stochastic variations at the level of individual patients. The impact of early diagnosis will be analysed using the updated Markov and

For peer review only

simulation models. With respect to patients detected earlier in a future screening protocol implemented in NOD, a key uncertainty is the stage of disease that would be diagnosed. A probabilistic sensitivity analysis, informed by the literature, will be conducted on a range of scenarios, including epidemiological, clinical and biomarker data.

Patient and public involvement statement

The Liverpool Pancreatic Patient and Public Involvement Group have contributed to the study conception and design and they have continued involvement in the management of this study. There are lay representatives on the Trial Steering Committee to ensure the study remains both acceptable and relevant to patients.

Ethics and Dissemination

Ethical approval

UK-EDI is approved by the UK Health Research Authority with favourable opinion granted by the London-West London and GTAC Research Ethics Committee on 14th February 2020. The details of this manuscript represent Version 5 of the protocol approved on the 9th September 2021.

The study will be conducted in accordance with the Human Rights Act 1998, the Data Protection Act 2018, Freedom of Information Act 2000, the principles of Good Clinical Practice, the Declaration of Helsinki on biomedical research involving human volunteers (Hong Kong revision, 1989 and the 48th General Assembly, Somerset West, Republic of South Africa, October 1996, updated in October 2013) and the UK Policy Framework for Health and Social Care Research. Where individuals agree to take part in the study, they will be informed of how data are recorded, collected, stored and processed, and that data may be transferred to other countries, in accordance with UK General Data Protection Regulations (UK GDPR).

Data Management considerations

For peer review only

Data Management will be through the Liverpool Clinical Trials Centre with delegated responsibilities for the University of Liverpool. The study has a dedicated Trial Manager, Data Manager and Trial Statistician and will be overseen by a Trial Management Committee and Trial Steering Committee. Data Management will be through the REDCap database and a dedicated Laboratory Information Management System (LIMS) within the Good Clinical Practice Laboratories at the University of Liverpool, linked by unique codes for each kit used at each time point from each patient, the code for which will be stored on REDCap and LIMS.

Dissemination plan

Study results will be disseminated through presentations at national and international symposia and publication in peer-reviewed, Open Access journals. Where appropriate data will be made available via open access repositories. We will work with charities, patient and public involvement groups, and other relevant stakeholders to widely disseminate results and ensure that our findings are in an accessible format.

Acknowledgements

The authors are grateful for the support of all participants contributing to this study.

Authors' contributions

EC, LO, WG, CH, RVDM, LA, RT, TP, DP and PG conceived and designed the study. LO, MS and EC drafted this manuscript. RH, MS, EC, WG, RJ, CH, WR and VC drafted the study protocol. RJ is responsible for statistical design and analysis. EC is chief investigator. All authors contributed to the final manuscript and agreed to all of the content of the submitted manuscript.

Funding statement

This work is supported by Cancer Research UK, grant number C7690/A26881.

Competing interests statement

EC, LO, WG, CH and PG are named as inventors on GB patent GBGB1806002.0; PCT/GB2019/050998, submitted by the University of Liverpool, that covers the measurement of

For peer review only

adiponectin and IL-1Ra as a biomarker for early detection of pancreatic cancer. No other competing interests declared.

References

1. Sung H, Ferlay J, Siegel RL, et al. Global Cancer Statistics 2020: GLOBOCAN Estimates of Incidence and Mortality Worldwide for 36 Cancers in 185 Countries. *CA: a cancer journal for clinicians* 2021;71(3):209-49. doi: 10.3322/caac.21660 [published Online First: 2021/02/05]
2. Office for National Statistics. Cancer survival in England 2013 - 2017. ONS website; 12 August 2019: Accessed June 2022
3. Neoptolemos JP, Palmer DH, Ghaneh P, et al. Comparison of adjuvant gemcitabine and capecitabine with gemcitabine monotherapy in patients with resected pancreatic cancer (ESPAC-4): a multicentre, open-label, randomised, phase 3 trial. *Lancet* 2017;389(10073):1011-24. doi: 10.1016/s0140-6736(16)32409-6 [published Online First: 2017/01/29]
4. Owens DK, Davidson KW, Krist AH, et al. Screening for Pancreatic Cancer: US Preventive Services Task Force Reaffirmation Recommendation Statement. *Jama* 2019;322(5):438-44. doi: 10.1001/jama.2019.10232 [published Online First: 2019/08/07]
5. Greenhalf W, Grocock C, Harcus M, et al. Screening of high-risk families for pancreatic cancer. *Pancreatology* 2009;9(3):215-22. doi: 10.1159/000210262 [published Online First: 2009/04/08]
6. Pereira SP, Oldfield L, Ney A, et al. Early detection of pancreatic cancer. *The Lancet Gastroenterology & Hepatology* 2020 doi: https://doi.org/10.1016/S2468-1253(19)30416-9
7. Pannala R, Leirness JB, Bamlet WR, et al. Prevalence and clinical profile of pancreatic cancer-associated diabetes mellitus. *Gastroenterology* 2008;134(4):981-87. doi: 10.1053/j.gastro.2008.01.039
8. Aggarwal G, Kamada P, Chari ST. Prevalence of diabetes mellitus in pancreatic cancer compared to common cancers. *Pancreas* 2013;42(2):198-201. doi: 10.1097/MPA.0b013e3182592c96 [published Online First: 2012/09/25]
9. Ose DJ, Viskochil R, Holowatyj AN, et al. Understanding the Prevalence of Prediabetes and Diabetes in Patients With Cancer in Clinical Practice: A Real-World Cohort Study. *Journal of the National Comprehensive Cancer Network : JNCCN* 2021;19(6):709-18. doi: 10.6004/jnccn.2020.7653 [published Online First: 2021/06/16]
10. Brewer MJ, Doucette JT, Bar-Mashiah A, et al. Glycemic Changes and Weight Loss Precede Pancreatic Ductal Adenocarcinoma by up to 3 Years in a Diverse Population. *Clin Gastroenterol Hepatol* 2022;20(5):1105-11.e2. doi: 10.1016/j.cgh.2021.07.046 [published Online First: 2021/08/07]
11. Mueller AM, Meier CR, Jick SS, et al. Characterization of the deterioration of diabetes control in patients with a subsequent diagnosis of pancreatic cancer: A descriptive study. *Pancreatology* 2022;22(3):387-95. doi: 10.1016/j.pan.2022.03.012 [published Online First: 2022/03/23]
12. Tan PS, Garriga C, Clift A, et al. Temporality of body mass index, blood tests, comorbidities and medication use as early markers for pancreatic ductal adenocarcinoma (PDAC): a nested case-control study. *Gut* 2022 doi: 10.1136/gutjnl-2021-326522 [published Online First: 2022/06/28]
13. Chari ST, Leibson CL, Rabe KG, et al. Pancreatic cancer-associated diabetes mellitus: Prevalence and temporal association with diagnosis of cancer. *Gastroenterology* 2008;134(1):95-101. doi: 10.1053/j.gastro.2007.10.040
14. Andersen DK, Korc M, Petersen GM, et al. Diabetes, Pancreatogenic Diabetes, and Pancreatic Cancer. *Diabetes* 2017;66(5):1103-10. doi: 10.2337/db16-1477 [published Online First: 2017/05/17]
15. Chari ST, Leibson CL, Rabe KG, et al. Probability of pancreatic cancer following diabetes: A population-based study. *Gastroenterology* 2005;129(2):504-11. doi: 10.1053/j.gastro.2005.05.007

16. Woodmansey C, McGovern AP, McCullough KA, et al. Incidence, Demographics, and Clinical Characteristics of Diabetes of the Exocrine Pancreas (Type 3c): A Retrospective Cohort Study. *Diabetes Care* 2017;40(11):1486-93. doi: 10.2337/dc17-0542 [published Online First: 2017/09/02]
17. Cui Y, Andersen DK. Pancreatogenic diabetes: special considerations for management. *Pancreatology* 2011;11(3):279-94. doi: 10.1159/000329188 [published Online First: 2011/07/16]
18. American Diabetes Association. 2. Classification and Diagnosis of Diabetes: Standards of Medical Care in Diabetes—2020. *Diabetes Care* 2019;43(Supplement_1):S14-S31. doi: 10.2337/dc20-S002
19. Hart PA, Kamada P, Rabe KG, et al. Weight Loss Precedes Cancer-Specific Symptoms in Pancreatic Cancer-Associated Diabetes Mellitus. *Pancreas* 2011;40(5):768-72. doi: 10.1097/MPA.0b013e318220816a
20. Vujasinovic M, Zaletel J, Tepes B, et al. Low prevalence of exocrine pancreatic insufficiency in patients with diabetes mellitus. *Pancreatology* 2013;13(4):343-6. doi: 10.1016/j.pan.2013.05.010 [published Online First: 2013/07/31]
21. Ewald N, Kaufmann C, Raspe A, et al. Prevalence of diabetes mellitus secondary to pancreatic diseases (type 3c). *Diabetes-Metabolism Research and Reviews* 2012;28(4):338-42. doi: 10.1002/dmrr.2260
22. Maitra A, Sharma A, Brand RE, et al. A Prospective Study to Establish a New-Onset Diabetes Cohort: From the Consortium for the Study of Chronic Pancreatitis, Diabetes, and Pancreatic Cancer. *Pancreas* 2018;47(10):1244-48. doi: 10.1097/MPA.0000000000001169 [published Online First: 2018/10/17]
23. Chari ST, Maitra A, Matrisian LM, et al. Early Detection Initiative: A randomized controlled trial of algorithm-based screening in patients with new onset hyperglycemia and diabetes for early detection of pancreatic ductal adenocarcinoma. *Contemporary clinical trials* 2022;113:106659. doi: 10.1016/j.cct.2021.106659 [published Online First: 2021/12/27]
24. Oldfield L, Evans A, Rao RG, et al. Blood levels of adiponectin and IL-1Ra distinguish type 3c from type 2 diabetes: Implications for earlier pancreatic cancer detection in new-onset diabetes. *eBioMedicine* 2022;75:103802. doi: https://doi.org/10.1016/j.ebiom.2021.103802
25. EuroQol Research Foundation. EQ-5D-5L User Guide, 2019; Available from: https://euroqol.org/publications/user-guides.2022. Accessed Sept 2022
26. Schmitt A, Gahr A, Hermanns N, et al. The Diabetes Self-Management Questionnaire (DSMQ): development and evaluation of an instrument to assess diabetes self-care activities associated with glycaemic control. *Health and Quality of Life Outcomes* 2013;11(1):138. doi: 10.1186/1477-7525-11-138

For peer review only

Figure legends

Figure 1: UK Early Detection Initiative for Pancreatic Cancer (UK-EDI) Programme outline. *NOD: new-onset diabetes mellitus; PDAC: pancreatic ductal adenocarcinoma; T2DM: type 2 diabetes mellitus; T3cDM: type 3c diabetes mellitus; WP: work package*

Figure 2: Schematic of UK-EDI study recruitment and participant follow-up. *HbA1c: glycated haemoglobin; EMIS: Egton Medical Information Systems (electronic healthcare record system)*

For peer review only - http://bmjopen.bmj.com/site/about/guidelines.xhtml

Word count (excluding abstract, figures and tables): 3281

For peer review only

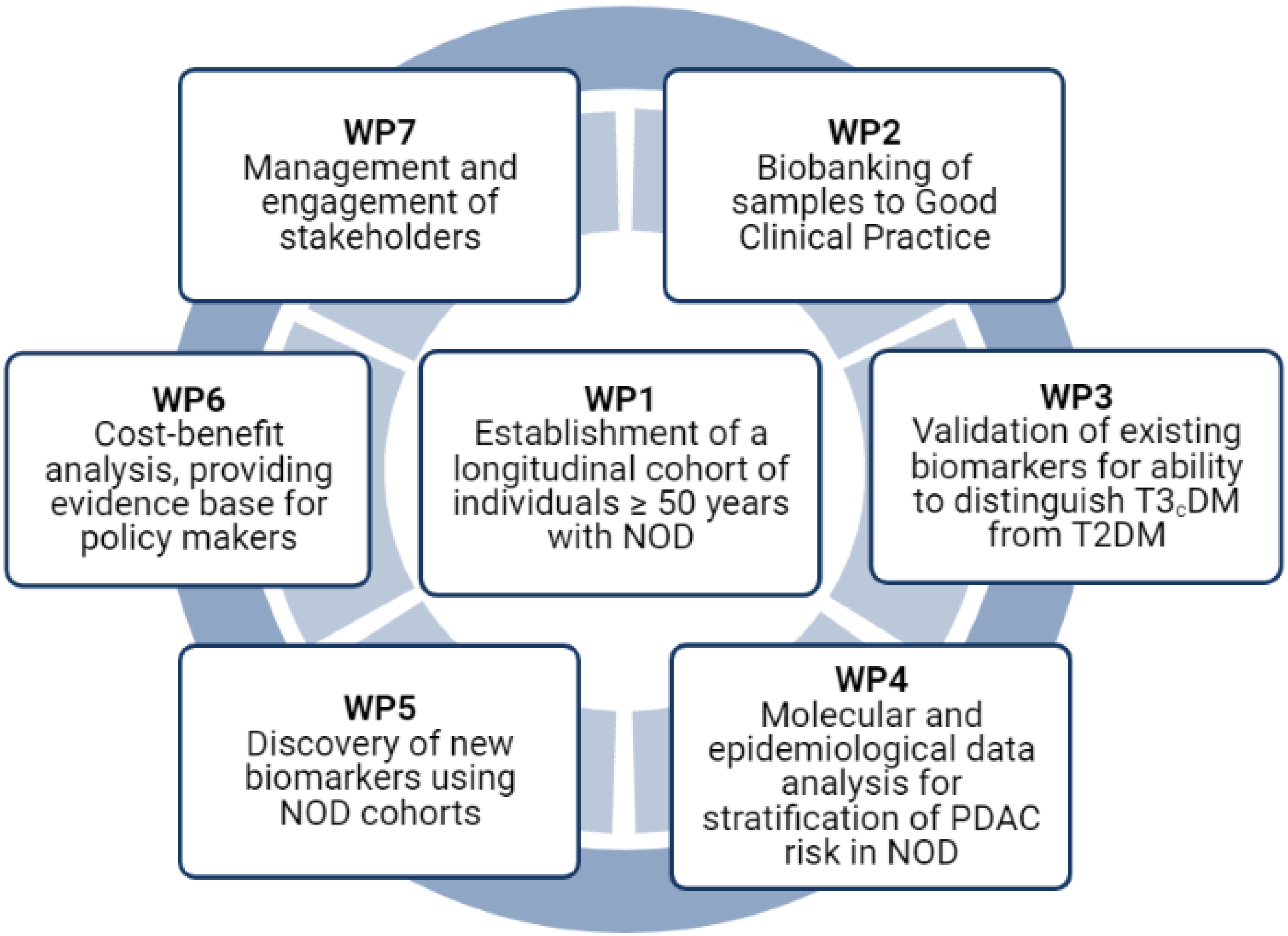
WP7
Management and engagement of stakeholders
WP2
Biobanking of samples to Good Clinical Practice
WP6
Cost-benefit analysis, providing evidence base for policy makers
WP1
Establishment of a longitudinal cohort of individuals ≥ 50 years with NOD
WP3
Validation of existing biomarkers for ability to distinguish $T3_cDM$ from T2DM
WP5
Discovery of new biomarkers using NOD cohorts
WP4
Molecular and epidemiological data analysis for stratification of PDAC risk in NOD

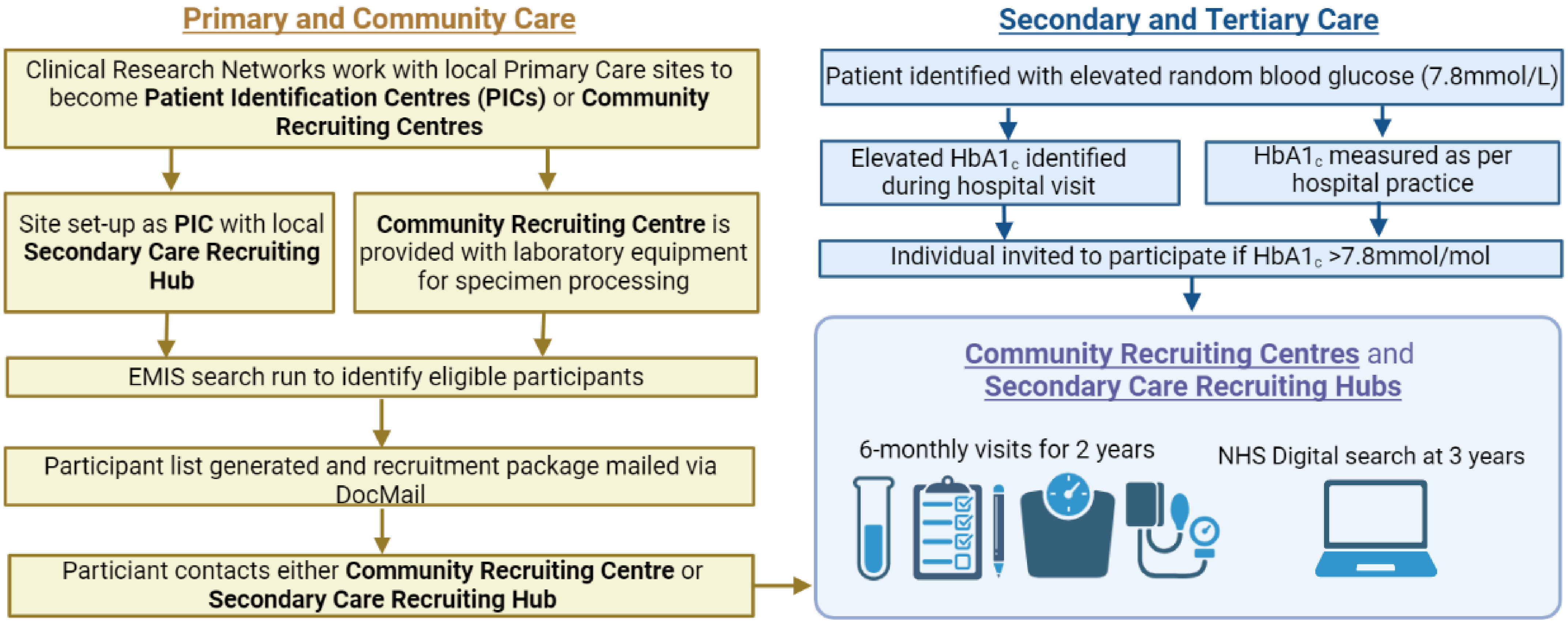
Primary and Community Care
Clinical Research Networks work with local Primary Care sites to become Patient Identification Centres (PICs) or Community Recruiting Centres
Site set-up as PIC with local Secondary Care Recruiting Hub
Community Recruiting Centre is provided with laboratory equipment for specimen processing
EMIS search run to identify eligible participants
Participant list generated and recruitment package mailed via DocMail
Particiant contacts either Community Recruiting Centre or Secondary Care Recruiting Hub
Secondary and Tertiary Care
Patient identified with elevated random blood glucose (7.8mmol/L)
Elevated HbA1c identified during hospital visit
HbA1c measured as per hospital practice
Individual invited to participate if HbA1c >7.8mmol/mol
Community Recruiting Centres and Secondary Care Recruiting Hubs
6-monthly visits for 2 years
NHS Digital search at 3 years
