# Supplementary material for: United Kingdom Early Detection Initiative (UK-EDI): protocol for establishing a national multicentre cohort of individuals with new-onset diabetes for early detection of pancreatic cancer
Source: BMJ Open. 2022 Oct 10;12(10):e068010. doi: 10.1136/bmjopen-2022-068010 (PMC9557307; doi:10.1136/bmjopen-2022-068010)
Supplement: Reviewer comments [file bmjopen-2022-068010.reviewer_comments.pdf]

PEER REVIEW HISTORY

BMJ Open publishes all reviews undertaken for accepted manuscripts. Reviewers are asked to complete a checklist review form (**http://bmjopen.bmj.com/site/about/resources/checklist.pdf**) and are provided with free text boxes to elaborate on their assessment. These free text comments are reproduced below.

ARTICLE DETAILS

**TITLE (PROVISIONAL)**	The United Kingdom Early Detection Initiative (UK-EDI): Protocol for establishing a national multi-centre cohort of individuals with new-onset diabetes for early detection of pancreatic cancer
**AUTHORS**	Oldfield, Lucy; Stott, Martyn; Hanson, Robert; Jackson, Richard; Reynolds, William; Chandran-Gorner, Vatshala; Van Der Meer, Robert; Alison, Laurence; Tejeiro, Ricardo; Purewal, Tejpal; Ghaneh, Paula; Palmer, Daniel; Greenhalf, William; Halloran, Chris; Costello, Eithne

This article was not externally reviewed at BMJ Open. Protocols that have been independently assessed prior to submission to BMJ Open are usually fast-tracked to publication on the grounds that further substantial changes will not be possible. This independent assessment will usually be external, independent review for both a substantial grant award from a non-commercial or government funder and ethics approval.
